# Increased B Cell-Activating Factor Promotes Tumor Invasion and Metastasis in Human Pancreatic Cancer

**DOI:** 10.1371/journal.pone.0071367

**Published:** 2013-08-06

**Authors:** Mitsuhito Koizumi, Yoichi Hiasa, Teru Kumagi, Hirofumi Yamanishi, Nobuaki Azemoto, Tetsuji Kobata, Bunzo Matsuura, Masanori Abe, Morikazu Onji

**Affiliations:** 1 Department of Gastroenterology and Metabology, Ehime University Graduate School of Medicine, Ehime, Japan; 2 Department of Immunology, Dokkyo Medical University School of Medicine, Tochigi, Japan; H. Lee Moffitt Cancer Center & Research Institute, United States of America

## Abstract

B cell-activating factor (BAFF) is a cytokine belonging to the tumor necrosis factor (TNF) superfamily. It has been reported that BAFF is elevated in patients with autoimmune pancreatitis and contributes to the malignant potential of blood cancers and solid tumors. In this study, clinical evidence of increased BAFF levels in patients with pancreatic ductal adenocarcinoma (PDAC) was obtained, and the roles and mechanisms of BAFF in PDAC were clarified in human tissues of PDAC and from *in vitro* data of PDAC cell lines. Serum levels of BAFF in patients with PDAC were significantly higher than in healthy subjects (p = 0.0121). Patients with UICC stage IV PDAC (T1-4, N0-1, M1) had significantly higher levels of serum BAFF compared to patients with PDAC (p = 0.0182). BAFF was remarkably expressed in infiltrating B lymphocytes surrounding pancreatic cancer in human pancreatic tissues, suggesting that BAFF may play a role in progression of pancreatic cancer. PDAC cell lines were cultured with human recombinant BAFF, and morphology and gene expression were analyzed; pancreatic cancer cells changed to a fibroblast-like morphology, and showed altered gene expression of E-cadherin, vimentin and Snail. These BAFF-induced changes reflect enhanced cell motility and invasion. BAFF-R-overexpressing cell clones confirmed the association between these BAFF-induced changes and epithelial-mesenchymal transition (EMT)-related genes. BAFF was elevated in patients with metastatic advanced PDAC and induced alterations in PDAC cells via regulation of EMT-related genes. Elucidation of the precise role and mechanism of control of BAFF may lead to new therapeutic approaches with the aim of improving pancreatic cancer survival.

## Introduction

Pancreatic cancer is one of the cancers with the poorest prognosis in humans. The 5-year survival rate of pancreatic cancer is only about 6% due to the difficulty in diagnosis in early clinical stages, as well as to frequent metastases [1], [2]. Recently, new therapeutic options have been reported; however, treatment options are limited and the response to chemotherapy remains low [3], [4]. Identification of novel targets for pancreatic cancer could improve prognosis.

It has recently been reported that B cell-activating factor (BAFF), a proinflammatory cytokine, is elevated in patients with autoimmune pancreatitis [5]. BAFF is a cytokine that belongs to a subset of the tumor necrosis factor (TNF) superfamily. In a previous experiment in which serum levels of BAFF were examined in patients with pancreatic cancer [5], patients with metastasis appeared to have increased levels of BAFF.

BAFF is a 285-amino acid peptide glycoprotein that is expressed as a transmembrane protein, and is secreted in a soluble form from various cell types (monocytes, dendritic cells, T lymphocytes, and B lymphocytes) [6]–[8]. It is known to be associated with survival and maturation of B lymphocytes. BAFF is a ligand for three receptors: BAFF-receptor (BAFF-R) [9]; transmembrane activator, calcium-modulator, and cyclophilin ligand interactor (TACI) [10]; and B cell maturation antigen (BCMA) [11]. Moreover, a protein similar to BAFF, named a proliferation-inducing ligand (APRIL) [12], may be a ligand of TACI and BCMA. Binding of BAFF or APRIL to those receptors can activate various signaling pathways, including the nuclear factor-κB (NF-κB) pathway [13]–[15]. It has been reported that BAFF and APRIL contribute to the malignant potential of blood cancers and solid tumors [16]–[18]. However, the roles of BAFF, APRIL, and their receptors in pancreatic cancer have not yet been elucidated.

In this study, clinical evidence of increased BAFF levels in patients with pancreatic ductal adenocarcinoma (PDAC) was obtained, and the role and mechanism of BAFF in PDAC was clarified from clinical evidence and from *in vitro* data from PDAC cell lines.

## Materials and Methods

### Patients and pancreas specimens

Serum samples were examined from 44 patients with PDAC and healthy age- and sex-matched subjects. For diagnosis of staging, the tumor node metastasis system of the Union for International Cancer Control (UICC) was used. All serum samples were stored at −80°C before use. Specimens of PDAC were obtained from patients who underwent surgery. Written informed consent was obtained from all enrolled participants. The study protocol conformed to the ethical guidelines of the 1975 Declaration of Helsinki, and was approved by the Institutional Review Board of Ehime University Hospital (Approval number: 1107003). This study involving human specimens was registered in the University Hospital Medical Information Network (UMIN) Clinical Trials Registry (registration number 000008654).

### Enzyme-linked immunosorbent assay for BAFF and APRIL

Serum levels of BAFF and APRIL in all subjects were assayed using an enzyme-linked immunosorbent assay kit (R&D Systems, Minneapolis, MN, USA for BAFF; BioVendor, Candler, NC, USA for APRIL) following the manufacturer's recommendations.

### Immunohistochemistry of PDAC specimens

Pancreatic tissues were fixed in formalin. Sections (3 µm thick) were cut from each block and adjacent sections were stained with standard hematoxylin and eosin (H&E) and immunohistochemical staining techniques. Paraffin-embedded samples were dewaxed and rehydrated, and then antigens were retrieved by autoclaving for 1 min at 125°C in EDTA buffer (pH 9.0). Endogenous peroxidase activity was inactivated by incubation with methanol containing 1% hydrogen peroxidase for 20 min. The sections were then incubated in 1% blocking serum for 30 min to reduce nonspecific reactions. For immunohistochemistry, the sections were incubated with the relevant primary antibody (Table S1) at 4°C overnight. The tissue sections were treated with peroxidase-labeled secondary antibody (Histofine Simplestain Max PO; Nichirei, Tokyo, Japan) for 1 h at room temperature, and incubated with Simple Stain DAB Solution (Nichirei). Photomicrographs were taken using a Nikon Microphot FXA with a Nikon Digital Camera DXM 1200 (Nikon, Tokyo, Japan).

For the immunofluorescence staining, mounted and formalin-fixed sections were used. Sections were incubated with the relevant primary antibody (Table S1) at 4°C overnight. After washing with phosphate-buffered saline, the sections were incubated with DyLight488-conjugated donkey anti-goat antibody for BAFF or BAFF-R, and with DyLight549-conjugated donkey anti-mouse antibody for CD20. The nuclei of the sections were stained with 4,6-diamidino-2-phenylindole (Wako, Osaka, Japan). Slides were imaged using a Zeiss Axioskop 2 Plus fluorescence microscope and captured by a Zeiss AxioCam HRc digital camera (Zeiss, Tokyo, Japan) and saved to a computer. The photographs stained for BAFF, BAFF-R and CD20 were merged to evaluate their locations in the cells.

### Cells and culture conditions

Four pancreatic cancer cell lines (PANC-1, BxPC-3, AsPC-1 and MIA PaCa-2 cells), which were initially generated from patients with PDAC, and Ramos cells were obtained from the American Type Culture Collection (ATCC, Manassas, VA, USA). The PANC-1 and MIA PaCa-2 cells were cultured in Dulbecco's modified Eagle's medium (DMEM; Life Technologies, Carlsbad, CA, USA) supplemented with 10% fetal bovine serum (Life Technologies) and 1% penicillin. BxPC-3, AsPC-1 and Ramos cells were cultured in RPMI-1640 supplemented with 10% fetal bovine serum and 1% penicillin. Microphotographs were obtained after various treatments on an inverted microscope (Olympus IX70, Olympus, Tokyo, Japan) equipped with an Olympus DP12 digital camera. After being serum starved for 24 h, cells were incubated with recombinant BAFF (Reliatech, Braunschweig, Germany) and recombinant TGF-β (R&D Systems) for 48 h. For neutralizing the BAFF, goat anti-BAFF-R antibody (R&D Systems) was used, and goat IgG antibody (R&D Systems) was used as a control antibody.

### RNA extraction, cDNA synthesis and real-time RT-PCR

The RNA was reverse transcribed using RT-PCR kits (Applied Biosystems, Foster City, CA, USA) with an oligo d(T)_16_ primer under standard conditions. Real-time PCR amplification was performed using a LightCycler 480 (Roche, Basel, Switzerland) and 2 µL of purified cDNA product, 0.5 µL of sense primer (10 pmol/ µL), 0.5 µL of antisense primer (10 pmol/ µl), 1 µL of LightCycler Fast Start DNA Master SYBR Green I (Roche), and 0.8 µL of MgCl_2_ (25 mmol/L) (experimental conditions and sequences of the primers used to amplify human genes are indicated in Table S2). Commercial glyceraldehyde phosphate dehydrogenase (GAPDH) primer sets and β-actin primer sets (Roche) were used for PCR amplification under the conditions recommended by the manufacturer. GAPDH served as an internal reference gene, and the relative change was calculated by relative quantification, applying the formula 2^−ΔΔCt^. Reaction products were separated on 2% agarose gels.

### Western blotting

For Western blotting, 20 µg of protein was applied to the lanes of 4% to 12% Bis-Tris Gels (Life Technologies), then blotted onto Immobilon-P membranes (Millipore, Bedford, MA, USA), and incubated with the relevant primary antibody (Table S1). Appropriate species-specific conjugated secondary antibody kits were commercially obtained (GE Healthcare, Tokyo, Japan). Proteins were detected using the ECL prime Kit or the ECL Kit (GE Healthcare) with an ImageQuant LAS 4000 system (GE Healthcare).

### Wound healing/scratch test

PANC-1 cells were seeded into 6-well plates and incubated for 24 h under a serum-starved condition. After confirming that a complete monolayer had formed, the monolayers were wounded by scratching lines with a plastic tip. The wells were then washed once to remove any debris, and observed and photographed under the microscope. Thereafter, the plates were incubated at 37°C under 5% CO_2_ for 24 h with the recombinant human BAFF (Reliatech), after which the cells were observed and photographed. Cells were visualized with an Olympus Model IX70 inverted microscope (Olympus) using a 4× objective. Images were captured with an Olympus DP12 a digital camera (Olympus). The distance that the cells had migrated was measured on the photomicrographs. The percent wounded area filled was calculated as follows: {(mean wounded breadth – mean remained breadth)/mean wounded breadth} × 100 (%) [19].

### Invasion assay

To investigate cell invasion, a 96-well cell invasion assay kit (Cultrex, Trevigen, Gaithersburg, MD, USA) was used according to the manufacturer's instructions.

### Establishment of human BAFF-R transfectant cell clones

Cell clones overexpressing BAFF-R were developed using a plasmid, which could express the BAFF-R gene and the G418-resistant gene (pBCMGS-BAFF-R) [20]. PANC-1 cells were transfected with pBCMGS-BAFF-R using Lipofectamin 2000 (Life Technologies), and the transfected cell clones were selected by incubation with G418 (1000 µg/mL, Life Technologies). Finally, four cell clones that over-expressed BAFF-R were established.

### Flow cytometric analysis

Flow cytometric analysis was performed for the stained PANC-1 cells and human BAFF-R transfected cell clones. BAFF-R was detected with primary antibody specific for the BAFF-R (Table S1) followed by an additional incubation with Alexa 488-conjugated secondary antibody for goat IgG (Abcam, Tokyo, Japan). Those stained cells were examined using a FACScalibur (Becton Dickinson, Franklin Lakes, NJ, USA) and analyzed with FlowJo software (TreeStar Corporation, Ashland, OR, USA).

### Statistical analysis

All statistical analyses were performed using JMP 8.0 (SAS Institute, Tokyo, Japan). Data expressed are means and standard error (SE) or means and standard deviation (SD). Differences were analyzed using the Student t-test, Wilcoxon test and χ^2^ test. Statistical significance was defined as p<0.05 based on a two-tailed test. Correlations between two variables were evaluated by using Pearson's coefficient of correlation, and p-values of <0.05 were considered to represent statistical significance.

## Results

### Serum levels of BAFF in patients with advanced PDAC

**Figure 1:**
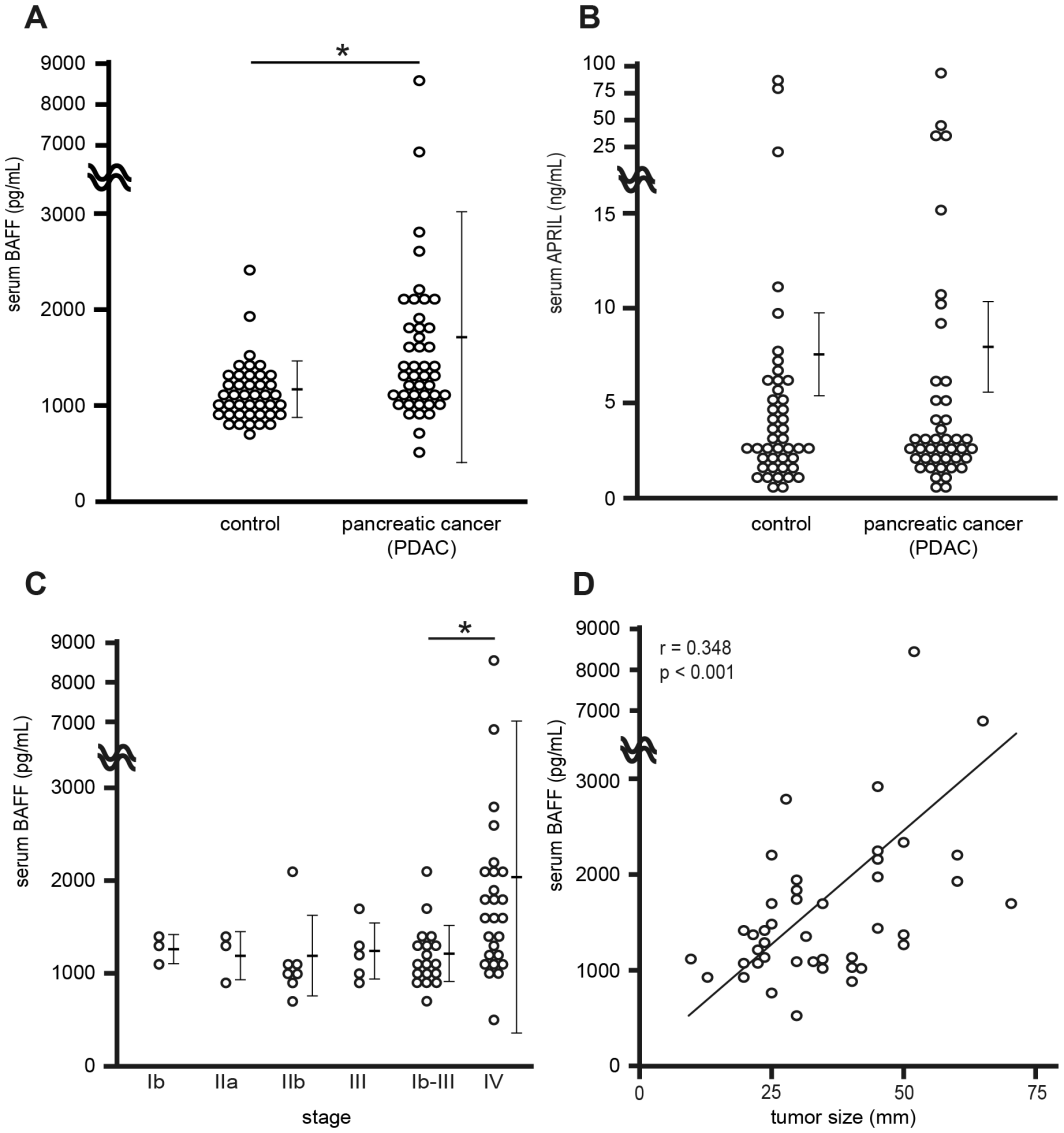
Serum levels of BAFF and APRIL in patients with PDAC. (A) Serum levels of BAFF were determined in patients with PDAC (n = 44) and in healthy subjects (n = 44). (B) Serum levels of APRIL were determined in patients with PDAC (n = 44) and in healthy subjects (n = 44). (C) Serum levels of BAFF and UICC stage of PDAC are indicated. (D) Serum levels of BAFF and tumor sizes of primary PDAC were significantly correlated in 44 patients (p<0.001). Data are shown as means ± SD (*p<0.05). n.s.; not significant.

Serum levels of BAFF and APRIL were examined in patients with PDAC and in healthy age- and sex-matched subjects (Table 1). Serum levels of BAFF were 1704 ± 1409 pg/mL (mean ± SD) in patients with PDAC, significantly higher than in healthy subjects (1147 ± 315 pg/mL; p = 0.0121) (Fig. 1A). On the other hand, serum levels of APRIL in patients with PDAC were not significantly higher than in healthy subjects (7.7 ± 2.4 vs. 7.3 ± 2.2 pg/mL, respectively; p = 0.895) (Fig. 1B). The increase in serum levels of BAFF was evaluated by UICC stage. Patients with UICC stage IV PDAC (T1-4, N0-1, M1) had significantly higher levels of serum BAFF than the patients with UICC stage Ib-III (2063 ± 1736 vs. 1186 ± 328 pg/mL, respectively; p = 0.0182) (Fig. 1C). The clinical background of the patients at each stage is indicated in Table 2. Concerning the relationship between the serum levels of BAFF and PDAC tumor size, there was a significant positive correlation, as indicated in Fig. 1D (r = 0.348; p<0.001). This suggested that the increase in serum levels of BAFF was associated with tumor growth and metastasis of PDAC.

**Table 1:** Clinical background of patients with pancreatic cancer and healthy subjects.

	Healthy subjects (n = 44)	PDAC (n = 44)	p-value
Age (years)	67 (40–92)	68 (40–89)	0.970
Gender (M/F)	26/18	26/18	1.000
BAFF (pg/mL)	1147 ± 315	1704 ± 1409	**0.0121**
APRIL (ng/mL)	7.3 ± 2.2	7.7 ± 2.4	0.895

Data are presented as numbers or means ± SD. PDAC, pancreatic ductal adenocarcinoma; BAFF, B cell-activating factor; APRIL, a proliferation-inducing ligand.

**Table 2:** Clinical background of patients with pancreatic ductal adenocarcinoma.

UICC stage	Total (n = 44)	Ib-III (n = 18)	IV (n = 26)	p-value
Age (years)	67.9 ± 11.6	67.1 ± 12.7	68.5 ± 11.4	0.713
Gender (M/F)	26/18	12/6	14/12	0.395
Location				0.050
Head	24	13	11	
Body and tail	20	5	15	
Tumor Size (mm)	35.5 ± 13.9	26.9 ± 9.9	41.5 ± 13.3	**<0.001**
Primary tumor (T)				**0.001**
T2	3	3	0	
T3	15	10	5	
T4	26	5	21	
Regional lymph nodes (N)				0.911
N0	20	8	12	
N1	24	10	14	
Distant metastasis (M)				**<0.001**
M0	18	18	0	
M1	26	0	26	
CEA (ng/m)	28.2 ± 83.7	5.0 ± 4.9	44.3 ± 106.6	0.0725
CA19-9 (U/mL)	9009 ± 40996	777 ± 1734	14709 ± 52974	0.192
BAFF (pg/mL)	1704 ± 1409	1186 ± 328	2063 ± 1736	**0.0182**
APRIL (ng/mL)	7.7 ± 2.4	3.9 ± 3.6	10.3 ± 18.9	0.0998

Data are presented as number or mean ± SD. UICC, International Union Against Cancer; CEA, carcinoembryonic antigen; CA19-9, carbohydrate antigen 19-9; BAFF, B cell-activating factor; APRIL, a proliferation-inducing ligand.

### Expression of BAFF and BAFF-R in human PDAC tissue

**Figure 2:**
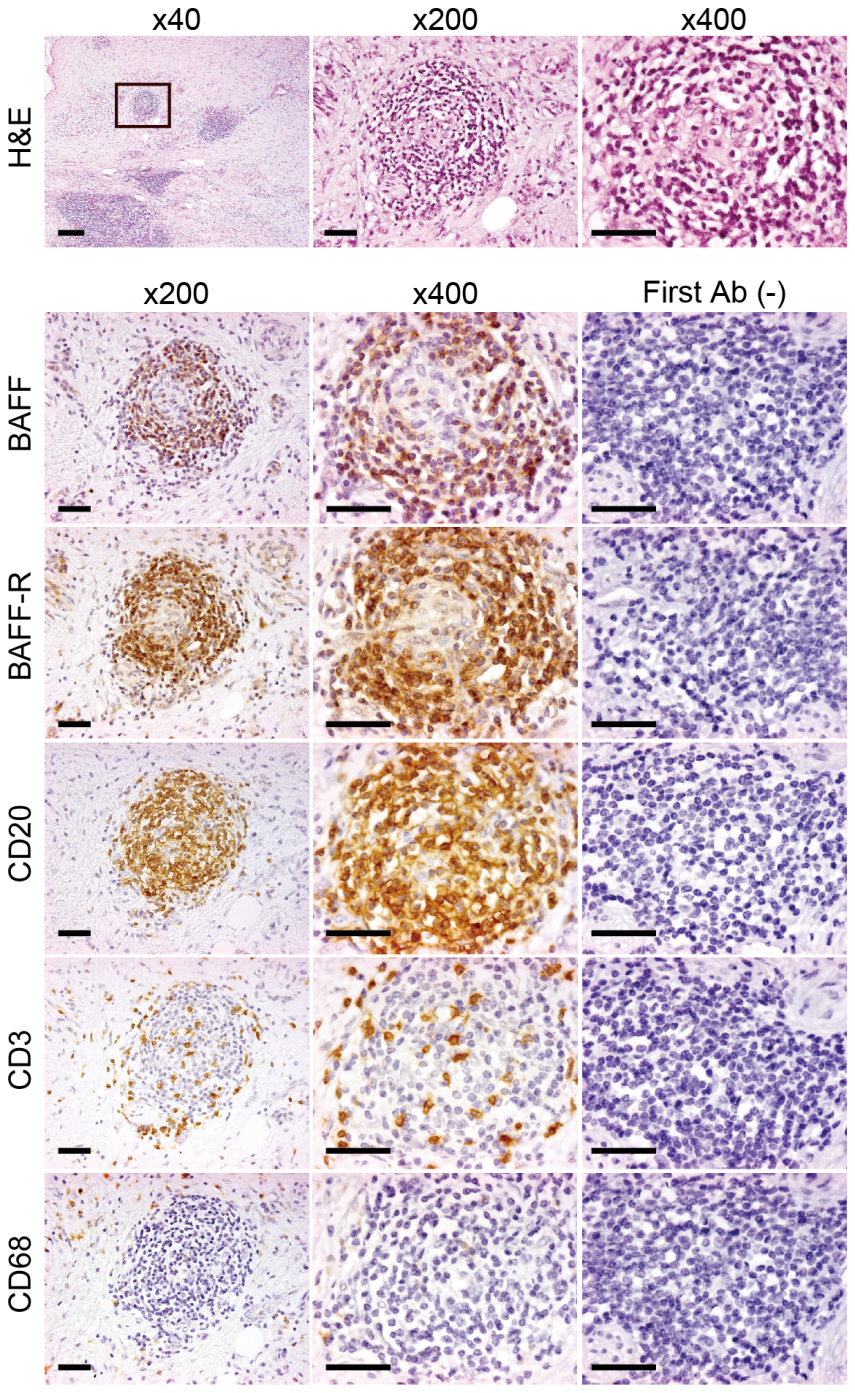
Immunohistochemistry of BAFF and BAFF receptor in human PDAC tissue. (A) Staining with hematoxylin and eosin (H&E) and immunohistochemistry of BAFF, BAFF-R, CD3, CD20 and CD68 in PDAC and surrounding tissues are shown. First Ab (-) indicates staining control without first antibody. The B lymphocytes in the tissue surrounding PDAC had a remarkably high level of BAFF expression. Magnification as indicated above each photomicrograph. Scale bars represent 200 µm under low magnification (x40), and 50 µm under high magnification (x200 and ×400).

**Figure 3:**
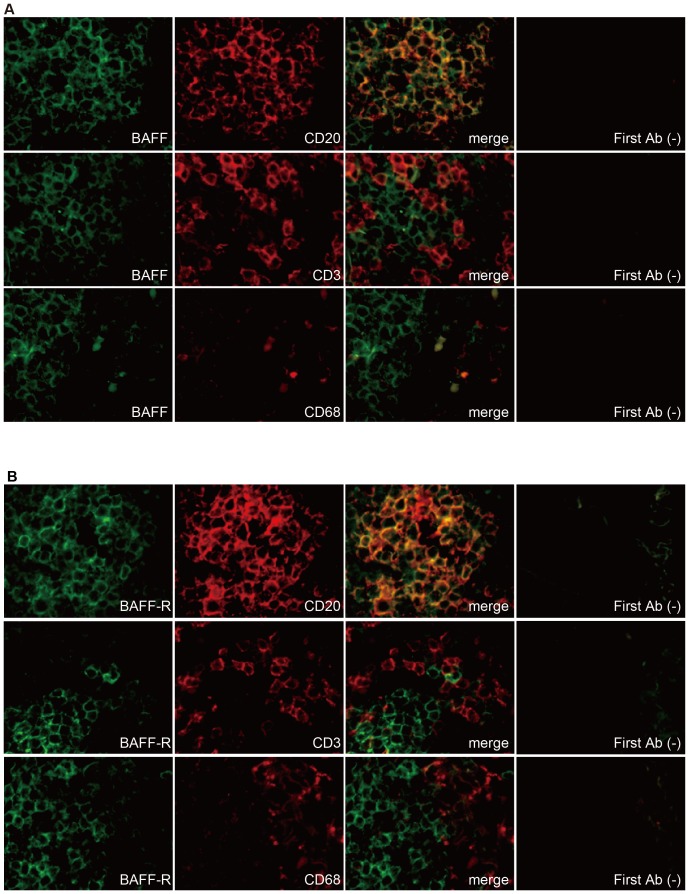
Immunofluorescence of BAFF and BAFF receptor in human PDAC tissue. (A) Immunofluorescence staining with BAFF (green), and CD20, CD3, CD68 (red) are shown. The merged image is indicated as “merge”. Expressions of BAFF and CD20 were co-localized (yellow); however, BAFF and CD3 or CD68 were not co-localized. (B) Immunofluorescence staining with BAFF-R (green) and CD20, CD3, and CD68 (red) are shown. Expression of BAFF-R and CD20 were co-localized (yellow); however, BAFF-R and CD3 or CD68 were not co-localized. First Ab (-) indicates staining control without the first antibody.

Immunohistochemistry experiments were performed to determine whether BAFF is expressed in pancreatic tissue. Pancreatic tissue from surgical species obtained from patients with PDAC was stained for BAFF and BAFF-R (Fig. 2 and 3). Tumor-infiltrating immune cells surrounding PDAC cells were found to remarkably express BAFF and BAFF-R (Fig. 2). Additional staining of CD20 (a marker of B lymphocytes), CD3 (T cell receptor, a marker of T lymphocytes) and CD68 (a marker of monocytes/macrophages) was performed to identify their types of infiltrating cells expressing BAFF or BAFF-R. The distribution of BAFF- and BAFF-R-positive cells appeared consistent with the distribution of B lymphocytes, rather than that of T lymphocytes or monocyte/macrophages (Fig. 2). Recently, it has been shown that B lymphocytes can secrete BAFF [7], [8], [16]. Double immunofluorescence staining with BAFF and CD20, CD3, CD68 (Fig. 3A), and staining with BAFF-R and CD20, CD3, CD68 (Fig. 3B) was performed. The distribution of BAFF (green) and CD20 (red) was merged and appeared yellow (Fig. 3A). Moreover, the distribution of BAFF-R (green) and CD20 (red) was also merged and appeared yellow (Fig. 3B). However, the distribution of BAFF and BAFF-R were not co-localized with CD3 or CD68. These results indicate that B lymphocytes, which express high levels of BAFF and BAFF-R, are in the infiltrate and proliferate in the tissue surrounding the PDAC.

### Expression of BAFF receptors in human PDAC tissues and human PDAC cell lines

**Figure 4:**
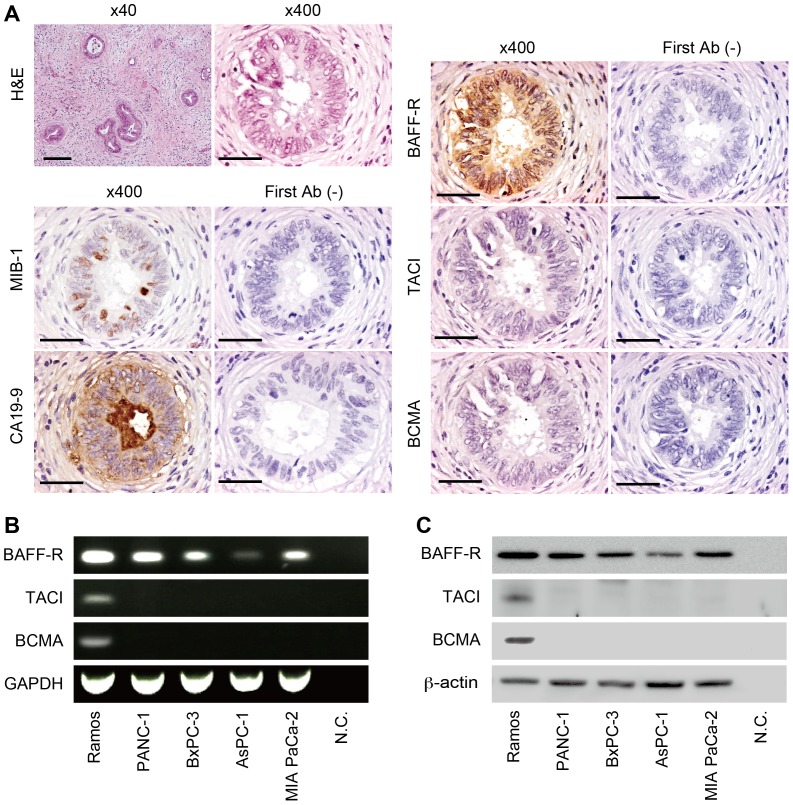
Expression of BAFF receptors in human PDAC tissue and human PDAC cell lines. (A) Staining with hematoxylin and eosin (H&E) and immunohistochemistry of MIB-1, CA19-9, BAFF-R, TACI and BCMA in PDAC are shown. First Ab (-) indicates staining control without the first antibody. Magnification is as indicated above each photomicrograph. Scale bars represent 200 µm under low magnification (x40), and 50 µm under high magnification (x400). (B) Qualitative RT-PCR analysis of BAFF receptors in PDAC cell lines. GAPDH is indicated as a housekeeping gene. RNA isolated from Ramos cell was used as a positive control. N.C. is a negative control without sample. (C) Western blot analysis of BAFF receptors in PDAC cell lines. β-actin is indicated as a housekeeping protein. Ramos cell was used as a positive control. N.C. is a negative control without sample.

In order to investigate the expression of BAFF receptors in PDAC, immunohistochemistry was performed on PDAC tissues obtained from patients as well as on human PDAC cell lines. BAFF-R was expressed in PDAC cells, which had positive signals of MIB-1 and CA19-9 (Fig. 4A, left column). However, other BAFF receptors (TACI and BCMA) were not expressed in the PDAC cells (Fig. 4A, right column). The human PDAC cell lines, PANC-1, BxPC-3, AsPC-1, and MIA PaCa-2, were used for the *in vitro* studies. Qualitative RT-PCR for the gene expression of BAFF-R, TACI, and BCMA revealed only expression of BAFF-R (Fig. 4B). Ramos cells, known as a tissue that expresses BAFF-R, TACI and BCMA, were used as a positive control [21]. Moreover, Western blotting was performed to evaluate the protein expression of these BAFF receptors (Fig. 4C). Expression of BAFF-R was confirmed in all of the PDAC cell lines, but expression of TACI and BCMA could not be detected. Taken together, these results showed that increased levels of BAFF resulted from the infiltrating and proliferating B lymphocytes surrounding PDAC tissues; these increased levels of BAFF can stimulate PDAC through BAFF-R signaling. To identify the role of BAFF in PDAC, an *in vitro* assay was performed using the PANC-1 PDAC cell line, its cloned transfectant of BAFF-R, and recombinant human BAFF for further analysis.

### BAFF induces EMT in a PDAC cell line

**Figure 5:**
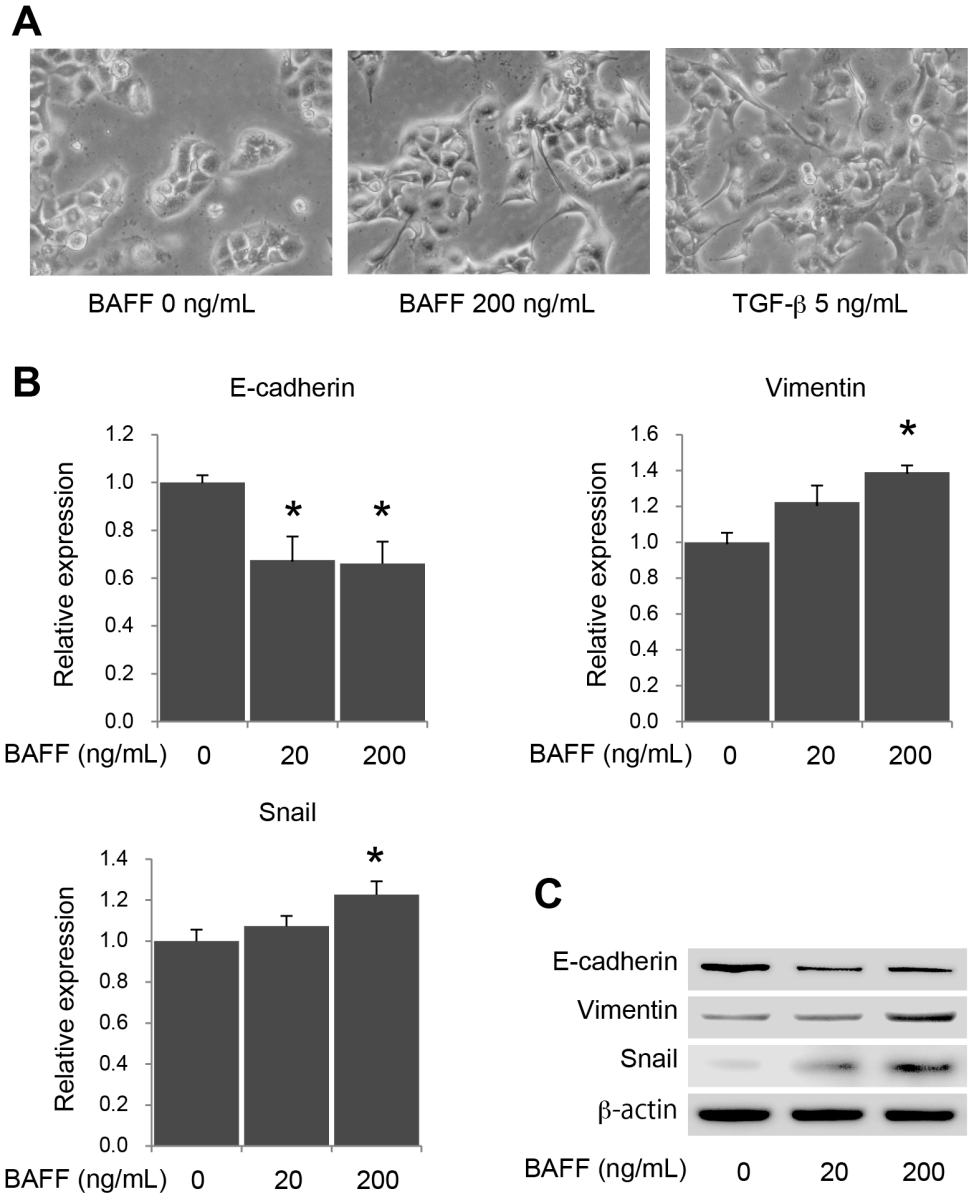
BAFF induces changes associated with epithelial-mesenchymal transition (EMT) in a PDAC cell line. (A) The cellular morphology of PANC-1 cells changed after the addition of BAFF to the supernatant. TGF-β was used as a positive control. Similar to TGF-β, addition of BAFF caused the morphology of PANC-1 cells to become spindle-like. (B) Analysis of real-time RT-PCR for EMT markers (E-cadherin, vimentin, and Snail) are indicated. These genes were modulated in a dose-dependent manner with BAFF. Data are shown as means ± SE of four separate experiments (*p<0.05 vs. BAFF 0 ng/mL). (C) Western blotting analysis of EMT markers indicates that the expression of each protein was modulated by the addition of BAFF. β-actin is shown as a housekeeping protein.

In the *in vitro* assay, the cell morphology of PANC-1 was observed to change after addition of recombinant human BAFF to the culture medium (Fig. 5A). The cells appeared to adopt a more fibroblast-like (spindle type) morphology and showed reduced cell-cell contact. These morphologic changes were similar to changes seen with TGF-β treatment. From this result, it was hypothesized that increased BAFF might induce alterations in genes related to the epithelial-mesenchymal transition (EMT) in PDAC cells. PANC-1 is isolated from undifferentiated PDAC tissue [22] and is positive for the epithelial marker E-cadherin and the mesenchymal protein vimentin. Thus, PANC-1 was used as a model of a PDAC cell that still has an epithelial potential. Analysis for altered expression of representative genes related to EMT was performed by adding human recombinant BAFF to the culture medium. A significant downregulation of E-cadherin mRNA, as well as significant upregulation of vimentin and Snail mRNAs, were observed in a dose-dependent manner with BAFF (Fig. 5B). GAPDH mRNA was used as a housekeeping gene for the quantitative PCR, and it was confirmed that the levels of GAPDH mRNA were not altered by treatment with BAFF (Fig. S1). Western blotting results for these molecules were similar to the results of real-time RT-PCR (Fig. 5C). The BAFF-induced alterations in PDAC cells were similar to alterations seen during EMT. Increased BAFF in PDAC could promote gene alterations associated with EMT through a decrease in E-cadherin and an increase in vimentin via the Snail signaling pathways.

### Treatment with human recombinant BAFF and motility and invasion of PDAC cells

**Figure 6:**
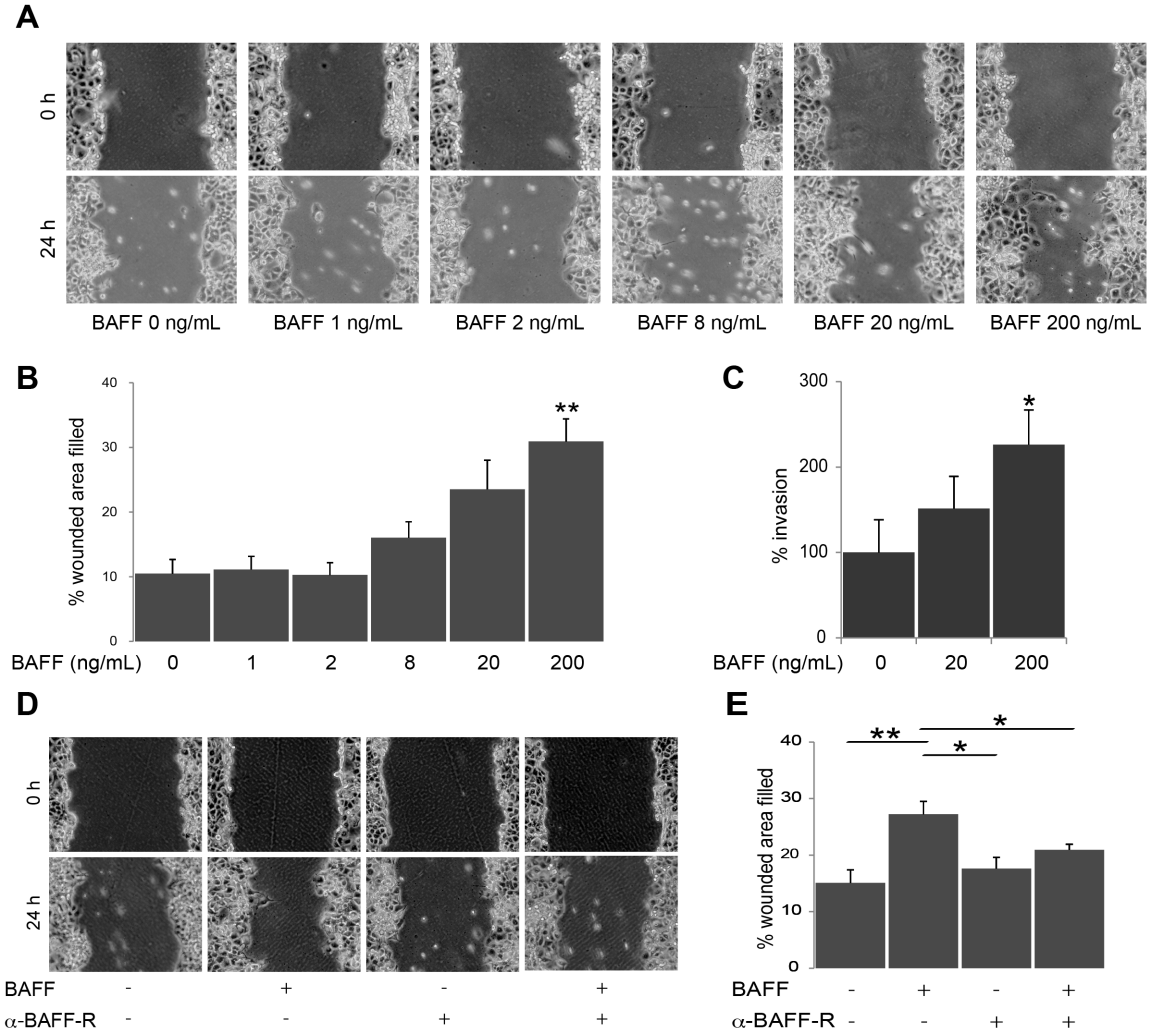
Treatment with human recombinant BAFF increases motility and invasion of PDAC cells. (A) The wound healing/scratch test indicated that the motility of PANC-1 cells was enhanced by 24 h of incubation with BAFF. (B) The percent wounded area filled in the wound healing/scratch test is shown as a graph (*p<0.01 vs. BAFF 0 ng/mL). (C) The invasion assay shows enhancement of invasion with BAFF in PANC-1 cells (*p<0.05 vs. BAFF 0 ng/mL). (D) The wound healing/scratch test with neutralizing anti-BAFF-R antibody indicated that the motility of PANC-1 cells with BAFF was inhibited by the antibody. (E) The percent wounded area filled in the wound healing/scratch test is shown as a graph. Data are shown as means ± SE of six separate experiments (*p<0.05, **p<0.01).

In conjunction with morphologic changes, it was suggested that BAFF induces EMT in PDAC. These results prompted study of the other effects of BAFF, such as motility and invasion. PANC-1 cells were incubated with human recombinant BAFF, and changes in motility were evaluated with the wound healing/scratch test. Treatment with BAFF increased the motility of PANC-1 cells and resulted in accelerated wound closure (p<0.01) (Fig. 6A and 6B). Moreover, treatment with human recombinant BAFF significantly improved the ability of cells to invade the extracellular matrix *in vitro* (Fig. 6C).

Additional assays were performed using anti-human BAFF-R antibody, which can inhibit the binding of BAFF to BAFF-R [23]. With addition of human recombinant BAFF to the supernatant of the cultured cells, the percent wounded area filled increased significantly (15.1 ± 2.3 vs. 27.2 ± 2.3; p<0.01). With addition of both human recombinant BAFF and the anti-human BAFF-R antibody, wound closure was diminished significantly compared to the addition of control goat antibody (27.2 ± 2.3 vs. 20.9 ± 2.4; p<0.05) (Fig. 6D and 6E). These data suggest that BAFF stimulation caused increased motility and invasion of PANC-1 cells.

### BAFF-R-overexpressing cell clones and expression of EMT-related genes

**Figure 7:**
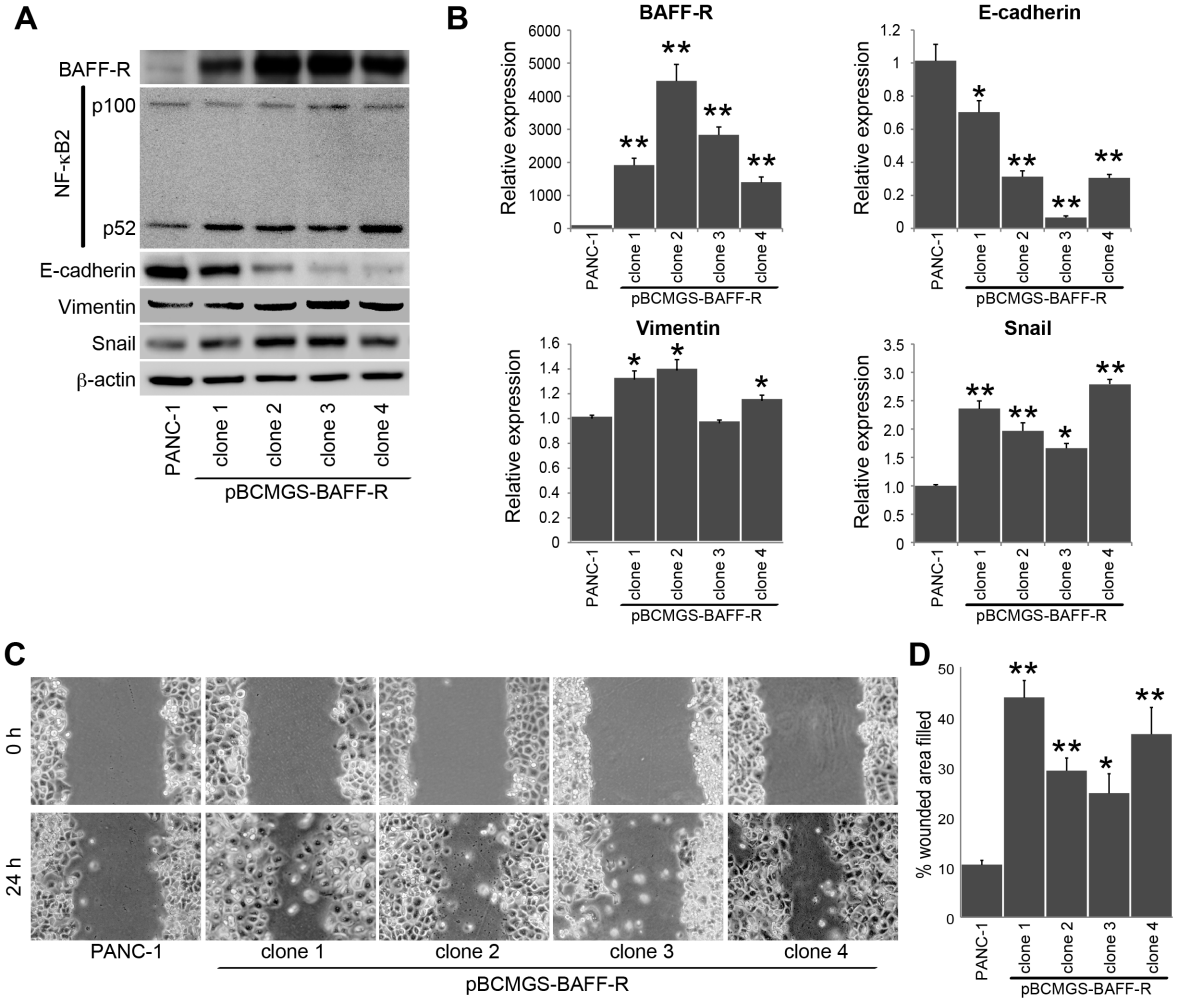
BAFF-R-overexpressing cell clones alter the expression of EMT-related genes. Four PDAC cell clones, which can overexpress BAFF-R through transfection with a plasmid (pBCMGS-BAFF-R), were established. (A) In the four clones, overexpression of BAFF-R was confirmed, and the proteins associated with EMT were found to be modulated in a BAFF-R level-dependent manner. (B) mRNA levels of each gene were evaluated with real-time RT-PCR. Data are shown as means ± SE of four separate experiments (*p<0.05, **p<0.01 vs. PANC-1). (C) The wound healing/scratch test indicated that the motility of these cell clones was enhanced compared to the original PANC-1. (D) The percent wounded area filled of each clone in the wound healing/scratch test is shown as a graph. Data are shown as means ± SE of six separate experiments (*p<0.05, **p<0.01 vs. PANC-1).

Four cell clones that can overexpress BAFF-R through transfection with a plasmid (pBCMGS-BAFF-R) [20] and selection by culturing with G418 were established. Overexpression of BAFF-R was confirmed in these cell clones by Western blotting and FACS analysis (Fig. 7A and S2). Functional signaling of transduced BAFF-R in these cell clones were confirmed by incubating with BAFF (Fig. S3). Increased expression of NF-κB p52 protein was seen on Western blotting, indicating that the NF-κB pathway was activated by overexpression of BAFF-R; this activation of the NF-κB pathway was also reflected in the decreased expression of E-cadherin and increased expression of vimentin and Snail in these clones. Those alterations were similar to the results of the assay with recombinant human BAFF.

Moreover, expression of mRNAs was evaluated using real-time RT-PCR (Fig. 7B). A decrease in E-cadherin mRNA was observed in all of the BAFF-R-overexpressing cell clones, while an increase in vimentin and Snail mRNAs was observed in most of the cell clones. Motility of cell clones was confirmed to be significantly higher than that of non-BAFF-R-transfected PANC-1 cells on the wound healing/scratch test (Fig. 7C and D). Taken together with the results from the cell clones, it was confirmed that stimulation with BAFF induces gene alterations associated with EMT in PDAC cells.

## Discussion

This is the first study to report that serum levels of BAFF are increased in patients with PDAC, and the first study to investigate the role of BAFF in human PDAC. In this study, it was revealed that: i) serum levels of BAFF in patients with PDAC (in particular, in those with metastasis) were elevated compared to healthy subjects; ii) tumor-infiltrating B lymphocytes expressed BAFF and PDAC tissues expressed BAFF-R; and iii) increased BAFF-induced gene alterations were associated with EMT in a PDAC cell line, and with enhanced tumor cell motility and invasion.

BAFF is known to be expressed by monocytes, dendritic cells, T lymphocytes, B lymphocytes, and epithelial cells [6]–[8], [24], [25]; however, its precise expression profile in patients with PDAC has not been previously defined. Recently, Nakajima et al. showed through immunohistochemistry that BAFF-expressing B lymphocytes infiltrate synovial tissues of rheumatoid arthritis [26]. The majority of infiltrating cells in the tissue surrounding PDAC in the present study were BAFF-expressing B lymphocytes. Moreover, many of these B lymphocytes also expressed BAFF-R. From these results, the infiltrating BAFF-expressing B lymphocytes could be considered to have an important role in the upregulation of BAFF in PDAC patients. The increased BAFF likely has a role in the progression of PDAC, because serum levels of BAFF were remarkably upregulated in the patients with advanced PDAC. BAFF produced from these B lymphocytes may also have an important role in the survival, activation, and proliferation of infiltrating B lymphocytes surrounding PDAC.

BAFF belongs to the TNF superfamily, and is closely related to APRIL. Both have an important role in the activation and proliferation of B lymphocytes. BAFF binds to all three BAFF receptors (BAFF-R, BCMA and TACI), whereas APRIL binds to two of them (BCMA and TACI). In the present study, only serum levels of BAFF (not APRIL) were significantly upregulated in patients with PDAC, and only the expression of BAFF-R could be detected in PDAC tissues. Thus, the role of BAFF and BAFF-R in PDAC was further explored. One of the most important BAFF-induced mechanisms is activation of NF-κB. It has been reported that BAFF-R activates NF-κB-inducing kinase (NIK), and that activation of NIK induces the processing of transcription factor NF-κB2 p100 to NF-κB2 p52 [27], [28]. The correlation between the activation of NF-κB and expression of Snail has previously been reported [29]. NF-κB appears to have an important role in the induction of EMT.

EMT is the phenomenon in which epithelial cells convert to mesenchymal cells; it is fundamental for embryonic development and involves profound phenotypic changes including loss of cell-cell adhesion and cell polarity and acquisition of migratory and invasive properties [30]. In previous reports, EMT has been characterized by the acquisition of a spindle-like/fibroblastic morphology, upregulation of mesenchymal markers like vimentin, and downregulation of epithelial marker like E-cadherin [31], [32]. Snail has been considered to be a trigger of EMT through downregulation of the expression of epithelial markers and upregulation of the expression of mesenchymal markers [33]. These molecules were upregulated by BAFF in PDAC cells; downregulation of E-cadherin and upregulation of vimentin were also observed in these cells.

**Figure 8:**
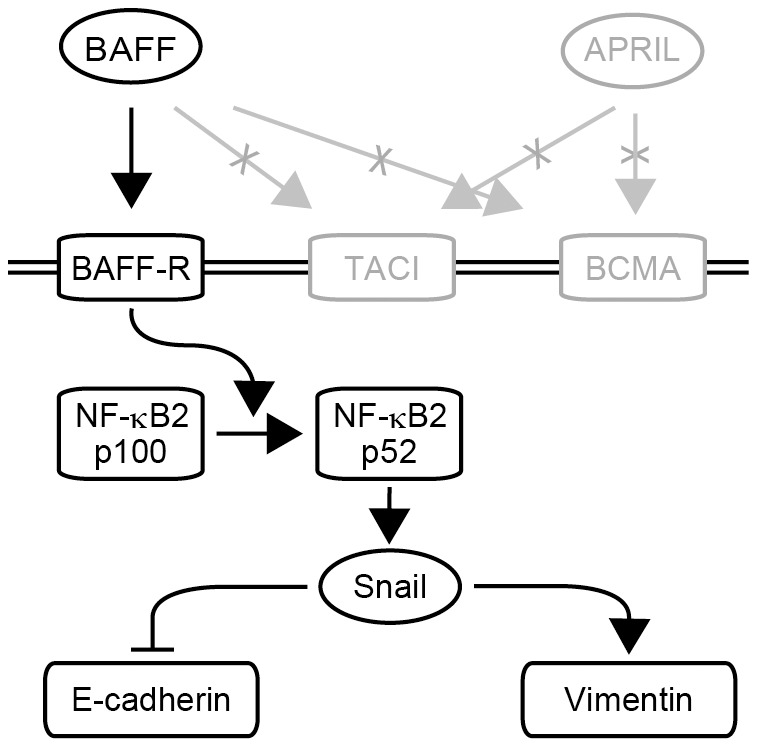
Model of the role of BAFF and the molecules associated with EMT. BAFF enhances cell motility and invasion through modulation of genes associated with EMT in PDAC.

From the present results, it is proposed that BAFF and BAFF-R signaling induces EMT in PDAC cells (Fig. 8). PDAC cells modulated by increased BAFF show a spindle-shaped morphology, lose cell-cell adhesion and cell polarity, and acquire the ability of cell motility and invasion. It is widely accepted that E-cadherin plays a critical role in EMT, an early event in cancer cell invasion and metastasis [34]. EMT is considered to be an important event during malignant tumor progression and metastasis [35], [36]. Therefore, these findings raise the possibility that infiltration of BAFF-expressing B lymphocytes increases the malignant potential of PDAC. Increased BAFF in tissue surrounding PDAC may provide a therapeutic target for controlling the progression of PDAC. Recently, an anti-BAFF antibody (Belimumab®) has been proposed for use in clinical studies against autoimmune diseases like rheumatoid arthritis [37]. Moreover, the anti-CD20 antibody (Rituximab®) was established for clinical use in patients with malignant B cell lymphoma and with autoimmune diseases such as rheumatoid arthritis [38], [39]. These drugs may also be effective in patients with advanced PDAC; however, for clinical use, further evaluation of their efficacy in animal models is needed.

In conclusion, it was found that serum levels of BAFF were elevated in patients with PDAC, particularly in patients with metastatic disease. BAFF-expressing B lymphocytes remarkably infiltrated the tissues surrounding the PDAC. Secreted BAFF may induce tumor progression and metastasis through modulation of EMT-associated genes. In the future, BAFF may be of use in clinical treatment designed to control the malignant potential of PDAC.

## Supporting Information

Figure S1 **The levels of GAPDH mRNA were not altered by the treatment of BAFF.** The levels of GAPDH mRNA were compared with the levels of β-actin mRNA over different doses of BAFF. The relative expression levels were not altered by the treatment of BAFF. Data are shown as means ± SE of four separate experiments.
(TIF)

Figure S2 **FACS analysis for the PANC-1 cells and human BAFF-R transfect cell clones with anti–BAFF-R antibodies.** PANC-1 cells and human BAFF-R transfect cell clones were stained with anti-BAFF-R antibodies. The blue line indicates the stained cells with anti-BAFF-R antibody, and the red line indicates controls.
(TIF)

Figure S3 **Functional signaling of transduced BAFF-R occurred in the BAFF-R-overexpressing cell clones.** Expression of NF-κB p52 was increased by adding 200 ng/mL of BAFF in each BAFF-R-overexpressing cell clone.
(TIF)

Table S1 **Antibodies used.**
(DOC)

Table S2 **Primer sequences used for real-time PCR.**
(DOC)
