# Supplementary figures and images for: Increased B Cell-Activating Factor Promotes Tumor Invasion and Metastasis in Human Pancreatic Cancer
Source: PLoS One. 2013 Aug 6;8(8):e71367. doi: 10.1371/journal.pone.0071367 (PMC3735500; doi:10.1371/journal.pone.0071367)

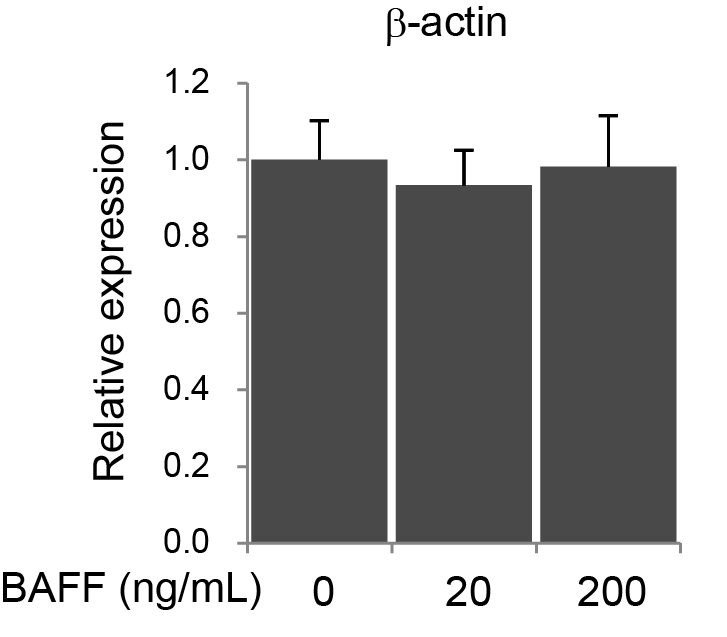

Supplement: Figure S1 — The levels of GAPDH mRNA were not altered by the treatment of BAFF. The levels of GAPDH mRNA were compared with the levels of β-actin mRNA over different doses of BAFF. The relative expression levels were not altered by the treatment of BAFF. Data are shown as means ± SE of four separate experiments. (TIF) [file pone.0071367.s001.tif]

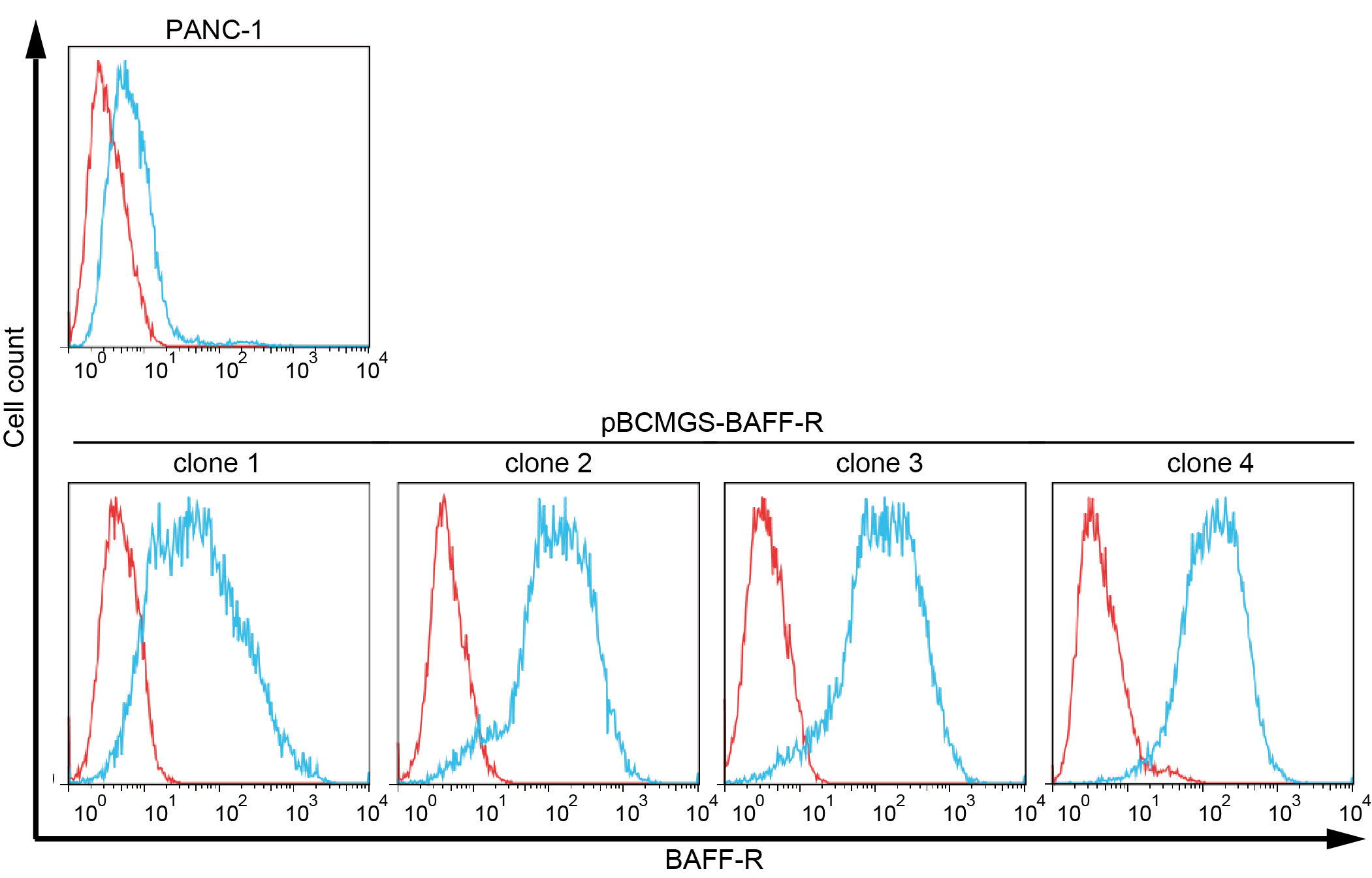

Supplement: Figure S2 — FACS analysis for the PANC-1 cells and human BAFF-R transfect cell clones with anti–BAFF-R antibodies. PANC-1 cells and human BAFF-R transfect cell clones were stained with anti-BAFF-R antibodies. The blue line indicates the stained cells with anti-BAFF-R antibody, and the red line indicates controls. (TIF) [file pone.0071367.s002.tif]

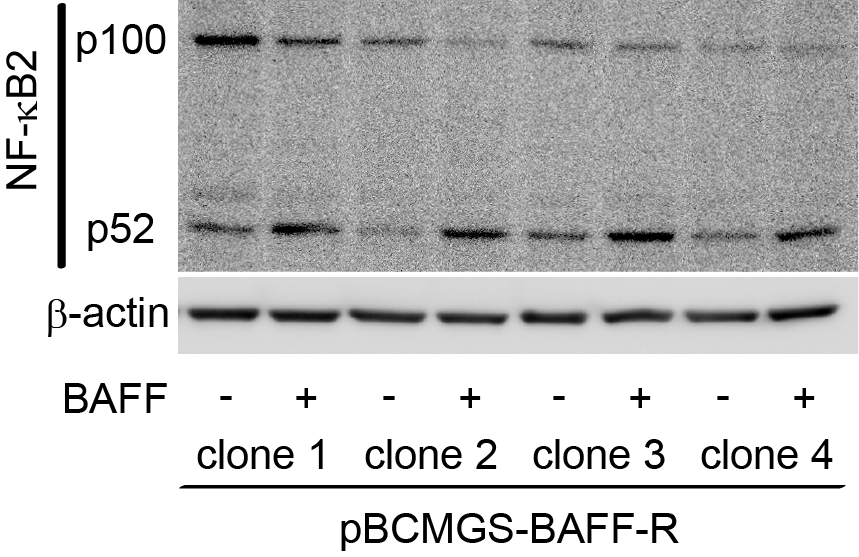

Supplement: Figure S3 — Functional signaling of transduced BAFF-R occurred in the BAFF-R-overexpressing cell clones. Expression of NF-κB p52 was increased by adding 200 ng/mL of BAFF in each BAFF-R-overexpressing cell clone. (TIF) [file pone.0071367.s003.tif]
