# Supplementary material for: Increased B Cell-Activating Factor Promotes Tumor Invasion and Metastasis in Human Pancreatic Cancer
Source: PLoS One. 2013 Aug 6;8(8):e71367. doi: 10.1371/journal.pone.0071367 (PMC3735500; doi:10.1371/journal.pone.0071367)
Supplement: Table S1 — Antibodies used. (DOC) [file pone.0071367.s004.doc]

**Supplemental Table 1.** Antibodies used

| Antibody | Company | Protocol |
| --- | --- | --- |
| BAFF | R&D Systems | IHC, IF |
| BAFF-R | R&D Systems | IHC, IF, WB, FCM |
| BAFF-R | Santa Cruz | WB |
| CD3 | Nichirei | IHC |
| CD20 | Dako | IHC, IF |
| CD68 | Dako | IHC |
| TACI | LifeSpan BioSciences | IHC |
| TACI | Imgenex | WB |
| BCMA | Enzo Life Sciences | IHC, WB |
| E-cadherin | Cell Signaling | WB |
| Vimentin | Cell Signaling | WB |
| Snail | Cell Signaling | WB |
| β-actin | Chemicon | WB |

IHC, Immunohistochemistry; IF, Immunofluorescence; WB, Western blotting; FCM, flow cytometry; BAFF, B cell-activating factor; BAFF-R, BAFF receptor; CD, cluster of differentiation; TACI, transmembrane activator, calcium-modulator, and cyclophilin ligand interactor; and BCMA, B cell maturation antigen.
