# Supplementary material for: Increased B Cell-Activating Factor Promotes Tumor Invasion and Metastasis in Human Pancreatic Cancer
Source: PLoS One. 2013 Aug 6;8(8):e71367. doi: 10.1371/journal.pone.0071367 (PMC3735500; doi:10.1371/journal.pone.0071367)
Supplement: Table S2 — Primer sequences used for real-time PCR. (DOC) [file pone.0071367.s005.doc]

**Supplemental Table 2. Primer sequences used for real-time PCR**

| Gene | Forward sequence | Reverse sequence | Annealing (^o^C) | Cycles |
| --- | --- | --- | --- | --- |
| BAFF-R | CTGGTCCTGGTGGGTCTG | ACCTTGTCCAGGGGCTCT | 72 | 35 |
| TACI | CAGCGGAGTGGAGAAGTTG | TGATCTGCACTCAGCTTCA | 60 | 30 |
| BCMA^1^ | GTCAGCGTTATTGTAATGCAAGTGT | TCTTTTCCAGGTCAATGTTAGCC | 68 | 35 |
| E-cadherin^2^ | CGGGAATGCAGTTGAGGATC | AGGATGGTGTAAGCGATGGC | 64 | 40 |
| Vimentin^3^ | GAGAACTTTGCCGTTGAAGC | TCCAGCAAGCTTCCTGTAGGT | 64 | 40 |
| Snail^4^ | GAGGCGGTGGCAGACTAG | GACACATCGGTCAGACCAG | 64 | 40 |

BAFF-R, BAFF receptor; CD, cluster of differentiation; TACI, transmembrane activator calcium-modulator, and cyclophilin ligand interactor; and BCMA, B cell maturation antigen.

Primers were designed as described previously.

^1^. Alexaki VI, Notas G, Pelekanou V, Kampa M, Valkanou M, Theodoropoulos P et al. Adipocytes as immune cells: differential expression of TWEAK, BAFF, and APRIL and their receptors (Fn14, BAFF-R, TACI, and BCMA) at different stages of normal and pathological adipose tissue development. [*J Immunol.*](http://www.ncbi.nlm.nih.gov/pubmed/19828625) 2009; **183**: 5948-5956.

^2^. Ohira T, Gemmill RM, Ferguson K, Kusy S, Roche J, Brambilla E et al. WNT7a induces E-cadherin in lung cancer cells. *Proc Natl Acad Sci* U S A. 2003; **100**: 10429-10434.

^3^. Tan BK, Chen J, Lehnert H, Kennedy R, Randeva HS. **Raised Serum, Adipocyte, and Adipose Tissue Retinol-Binding Protein 4 in Overweight Women with Polycystic Ovary Syndrome: Effects of Gonadal and Adrenal Steroids. *J Clin Endocrinol Metab*** 2007; **92**: 2764-2772**.**

^4^. Hotz B, Arndt M, Dullat S, Bhargava S, Buhr HJ, Hotz HG. Epithelial to mesenchymal transition: expression of the regulators snail, slug, and twist in pancreatic cancer. *Clin Cancer Res* 2007; **13**: 4769-4776.
